# Spontaneous Knee Ankylosis through Heterotopic Ossification after Total Knee Arthroplasty

**DOI:** 10.1155/2016/3548512

**Published:** 2016-04-07

**Authors:** Samuel Boulezaz, Emmanuel Gibon, Philippe Loriaut, Laurent Casabianca, Romain Rousseau, Benjamin Dallaudiere, Hugues Pascal-Moussellard

**Affiliations:** ^1^Department of Reconstructive and Orthopaedic Surgery, Clinical Orthopaedic Research Centre, Université Paris Descartes, Hôpital Cochin, 27 rue du Faubourg Saint Jacques, 75014 Paris, France; ^2^Department of Orthopaedic and Sport Surgery, Pitié Salpêtrière Hospital, Pierre and Marie Curie University, 47 boulevard de l'Hôpital, 75013 Paris, France; ^3^Centre d'Imagerie Ostéo-Articulaire, Clinique du Sport de Bordeaux-Mérignac 2, rue Négrevergne, 33700 Mérignac, France; ^4^Département d'Imagerie Musculo-Squelettique, Centre Hospitalier Universitaire Pellegrin, Place Amélie Léon Rabat, 33000 Bordeaux, France

## Abstract

This paper reports on a case of total ankylosis of the knee after a cruciate-sacrificing cemented total knee arthroplasty (TKA). An 82-year-old female patient previously underwent primary TKA for osteoarthritis twenty years ago in our institution. She had recovered uneventfully and returned to her regular activities. There was no history of postsurgical trauma; however, she progressively lost knee range of motion. Radiographs revealed severe bridging heterotopic ossification.

## 1. Introduction

Heterotopic ossification (HO) is the abnormal formation of mature lamellar bone in soft tissues and is a common finding in total hip arthroplasty; however, it is an unusual phenomenon after total knee arthroplasty (TKA). An incidence of 1% to 42% has been reported in the literature [1–8].

HO after TKA rarely results in complications. Most patients are asymptomatic, but some have reported pain or limited range of motion. HO leading to a bony ankylosis after TKA is very uncommon. Very few cases of total bony ankylosis after TKA have been reported, most of which had a history of previous sepsis [1, 4, 9–12].

The purpose of this case report is to describe the clinical and radiological features of spontaneous knee ankylosis by HO following primary TKA for osteoarthritis.

## 2. Case Report

In November 1994, a 62-year-old woman underwent a cemented left TKA in our institution using a G2S (FII, Saint-Etienne, France) posterior stabilized total knee prosthesis for primary osteoarthritis. Her only reported medical comorbidities at this time included hypertension (treated) and an increased body mass index (BMI) of 29.3 (75 kg, 160 cm).

Preoperatively, the patient was very disabled by her knee pain. Her walking tolerance was less than a hundred meters. She had been using analgesia and had received several knee infiltrations that were of limited efficacy prior to surgical management. The reported range of motion of the affected knee was 90/10/0 preoperatively. Her TKA was performed without a tourniquet and through a medial parapatellar approach.

Her infrapatellar fat pad was partially resected. As there was no major frontal deviation there was no need for ligament release. All the implants (tibial, femoral, and patellar) were cemented. No complications were reported during surgery.

Postoperatively, there was no malalignment or malposition on the X-rays, and she recovered uneventfully. Reported range of motion to her knee was 100/10/0 at 6-week follow-up. No further clinical data was available for this patient as she returned to her country of origin.

**Figure 1:**
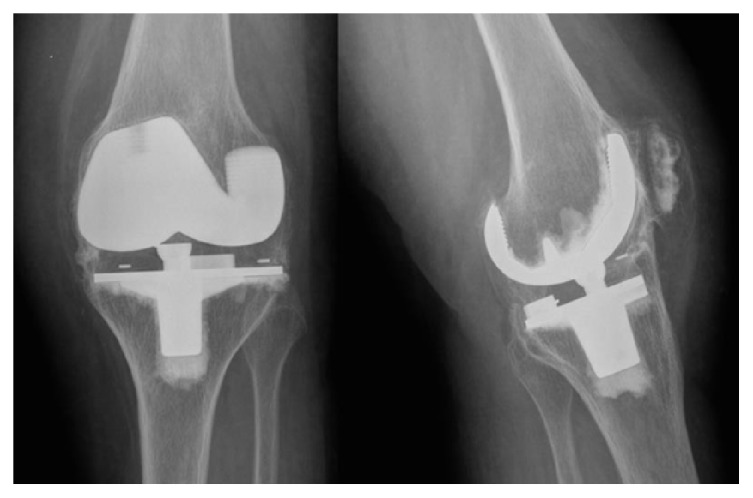
AP and lateral X-rays showing massive heterotopic ossification around the implants.

She returned to the clinic 20 years later, at 82 years of age. Clinical examination revealed a knee locked in full extension and quadriceps atrophy. The patient had no significant trauma history and reported no knee pain. Global function and autonomy were preserved. X-ray radiographs displayed massive circumferential and complete extra-articular ossifications around her prosthesis. The ossifications were located in both the femorotibial joint space and the femoropatellar joint. There was neither intra-articular calcification nor joint effusion (Figure 1).

**Figure 2:**
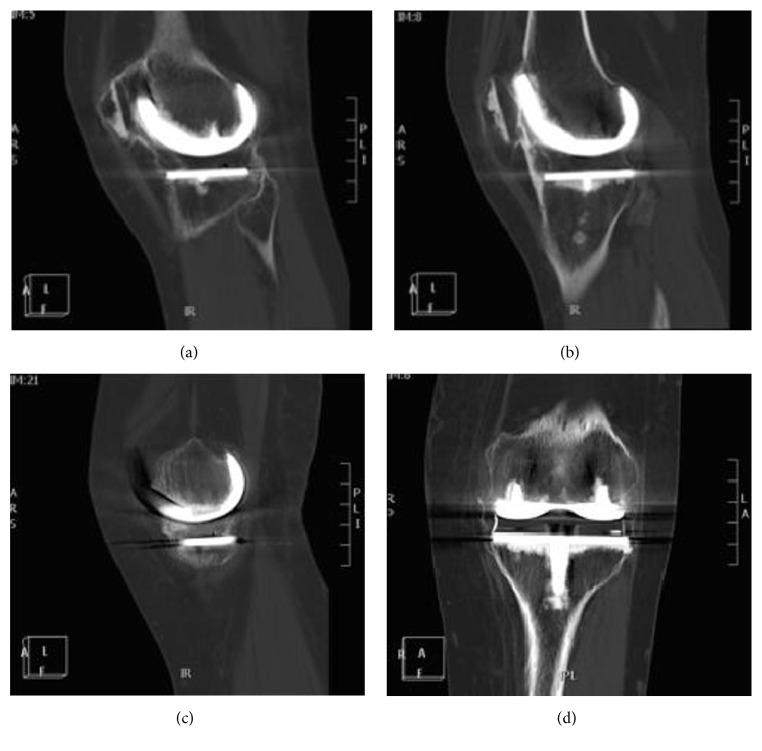
CT scan views showing a circumferential complete ankylosis of the knee.

**Figure 3:**
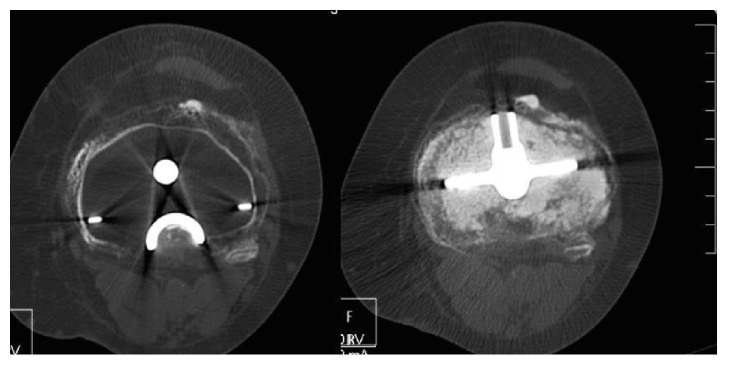
No lysis between cement and cancellous bone.

**Figure 4:**
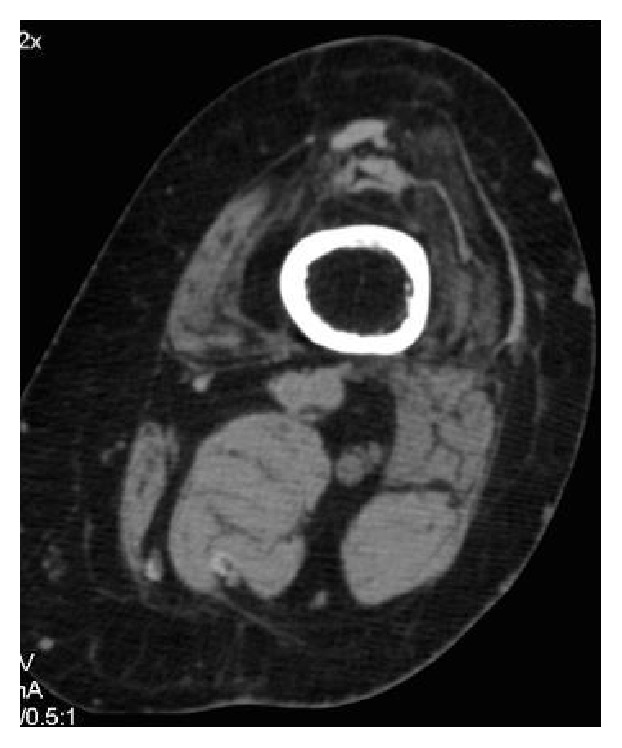
Complete quadriceps amyotrophy.

A CT scan confirmed the above observations and showed complete circumferential ankylosis with an absence of osteolysis around the TKA with a posterior joint fusion (Figures 2 and 3). Cancellous bone weft was with aspect of demineralized age related (82 years old) with no malignant or Paget disease signs. A complete quadriceps amyotrophy was observed (Figure 4). There was neither fibrocartilage calcification nor hyaline cartilage lesions suggesting chondrocalcinosis or gout. No abarticular deformation or sign of renal osteodystrophy was observed.

In the 20 years where there was no documented orthopedic follow-up, the patient did not recall any trauma, infiltration, or infection in her knee. The scar remained clean.

Final radiological diagnosis was spontaneous total knee ankylosis with HO. Indeed, this complete knee ossification could be compared to dystrophic ossification leading to ankylosis after total hip arthroplasty.

Investigations focused on etiological research. Blood tests and blood cultures showed no evidence of infection or an inflammatory syndrome. Nevertheless, the hypothesis of a chronic infection was explored.

A CT-guided articular puncture was consequently performed and cultures were negative. Further surgery was offered to the patient, but she declined.

## 3. Discussion

This case report provides insight on a very rare situation of total knee ankylosis after TKA showing that HO may severely alter knee range of motion.

HO can be detected on a bone scan as early as three weeks postoperatively, with increased uptake in the soft tissues. Plain radiographs are negative for four to six weeks. However, the increased bone turnover that occurs in HO can be detected as early as one week after surgery, with excessive increase in the specific osteoclastic and osteoblastic markers (CTX-1 and P1NP) detected in venous blood. Extensive bone formation may occur within three months, but full maturation of bone takes up to one year [3, 13].

Risk factors of HO around the knee have been widely studied. PCL reconstruction in multiligament knee injuries [14], long-term sedation [15], dislocation, high Injury Severity Score, and closed head injury [16] have been found to be risk factors in native knee. Osteoarthritis, wound healing problems, hypertrophic arthritis, notching [17], elevated BMI, male gender [18], preoperative grade of osteophyte formation [19], limited postoperative knee flexion, excessive periosteal trauma, postoperative forced manipulation [20], postoperative effusion [21], and increased lumbar bone mineral density [5] have been found to be risk factors in total knee arthroplasty. The patient in this case presented with only limited postoperative knee flexion and an elevated BMI in her risk factor profile.

Several treatment options for symptomatic HO have been discussed in the literature. Physical therapy is the main recommended treatment [22]. Some authors have proposed surgical excision of large symptomatic HO while others suggested resection with concurrent manipulation, followed by postoperative indomethacin for 2 months and physical therapy [21–23].

Several preventive measures have been discussed in the literature. Irradiation in the prevention of HO has been discussed but is controversial as it carries significant side effects. Nonsteroidal anti-inflammatories (NSAIDs) such as indomethacin have inconclusive results and increase the risk of major bleeding [3, 24, 25].

A simple but effective measure is to wash the joint with serum during the surgical procedure. This should reduce the presence of bone particles in periarticular soft tissues.

## 4. Conclusion

Complete ankylosis after total knee arthroplasty is a rare but significant complication; physicians who care for arthroplasty patients should be made aware of.
